# LNGFR targets the Wnt/β-catenin pathway and promotes the osteogenic differentiation in rat ectomesenchymal stem cells

**DOI:** 10.1038/s41598-017-11555-9

**Published:** 2017-09-08

**Authors:** Gang Li, Junyu Liu, Yingying Wang, Kun Yang, Manzhu Zhao, Yong Xiao, Xiujie Wen, Luchuan Liu

**Affiliations:** 1 Department of Stomatology, Daping Hospital, Research Institute of Field Surgery, Third Military Medical University, Chongqing, 400042 China; 2 Department of Periodontology, Stomatological Hospital, Zunyi Medical College, Zunyi, Guizhou 563003 China

## Abstract

Considerable evidence has shown that the Wnt/β-catenin pathway is involved in osteogenic differentiation in various stem cells. However, the role of Wnt/β-catenin pathway in regulating the osteogenic differentiation of rat ectomesenchymal stem cells (EMSCs), which are considered to be the progenitors of dental mesenchymal stem cells, remains unknown. In this study, we demonstrated that nuclear β-catenin was upregulated during EMSC osteogenic differentiation. The Wnt signalling inhibitor IWR-1-endo inhibited EMSC osteogenic differentiation, while the Wnt signalling agonist SKL2001 promoted it. Moreover, nuclear β-catenin was further upregulated by the overexpression of low-affinity nerve growth factor receptor (LNGFR) during EMSC osteogenic differentiation. Further experiments demonstrated that LNGFR overexpression enhanced EMSC osteogenic differentiation, while LNGFR silencing decreased it. Additionally, IWR-1-endo attenuated LNGFR-enhanced EMSC osteogenic differentiation. Collectively, our data reveal that LNGFR targets the Wnt/β-catenin pathway and positively regulates EMSC osteogenic differentiation, suggesting that Wnt/β-catenin pathway may be involved in the development of teeth and that the targeting Wnt/β-catenin pathway may have great potential for applications in dental tissue engineering regeneration.

## Introduction

Ectomesenchymal stem cells (EMSCs), which are isolated from rat embryonic facial processes, are derived from the cranial neural crest^1^. At an early stage of embryogenesis, cranial neural crest-derived cells migrate to the maxillary and mandibular processes and are then defined as EMSCs^2^. After migration, EMSCs interact with the dental epithelium and subsequently differentiate into several mesenchymal stem cells, such as dental papilla and dental follicle cells, which in turn form the dentin, pulp, cementum and periodontal ligaments^3^. Therefore, EMSCs derived from the cranial nerve crest are considered to be the progenitors of dental mesenchymal stem cells^2^. Recently, emerging studies have reported that EMSCs are capable of osteogenic differentiation *in vitro*. Yan *et al*. reported that mouse EMSCs isolated from eight-week-old BALB/c mice embryos are capable of extensive multilineage differentiation *in vitro*, including osteogenic differentiation^4^. Bruno *et al*. reported that EMSCs isolated from human olfactory mucosa originated from neural crest origins and have the potential for osteogenic differentiation^5^. Our previous studies showed that EMSCs had multi-directional differentiation potential and could be used as a powerful cell model for studying tooth development *in vitro* ^1^. Investigating the mechanisms involved in the differentiation of EMSCs is of great significance for improving our understanding of tooth development and may have potential applications in periodontal/dental regeneration and tissue-engineering methods to repair orofacial structures.

The Wnt/β-catenin pathway is the canonical Wnt signalling pathway, and results in the accumulation of β-catenin in the cytoplasm and its final translocation into the nucleus as a transcriptional coactivator of transcription factors belonging to the TCF/LEF family^6^. Without Wnt signalling, β-catenin cannot accumulate in the cytoplasm because of the destruction complex. Once Wnt signalling is activated, the destruction complex function is destroyed, which allows β-catenin to accumulate and localize to the nucleus and ultimately upregulate the expression of Wnt/β-catenin target genes^7^. The Wnt/β-catenin pathway plays key roles in embryonic development, including in body axis patterning, cell fate specification, cell proliferation and cell migration^8^. Emerging studies have reported that the canonical Wnt/β-catenin pathway is closely associated with osteogenic differentiation in tooth development. For example, Han *et al*. reported that activation of the canonical Wnt signalling pathway significantly increased mineralization, alkaline phosphatase activity, and protein expression of bone and cementum markers in human periodontal ligament cells^9^. Liang *et al*. reported that the Wnt/β-catenin signalling pathway contributes to the ET-1-enhanced differentiation of periodontal ligament stem cells into osteoblasts under an inflammatory microenvironment^10^. Mao *et al*. reported that the canonical Wnt signalling inhibitor dickkopf1 repressed the mnHA bioceramics-induced upregulation of ALP activity and osteogenic and cementogenic gene expression in human periodontal ligament stem cells^11^. Moreover, Liu *et al*. reported that the telomerase reverse transcriptase/Wnt/β-catenin cascade is upregulated by acetylsalicylic acid treatment, leading to improved stem cells from exfoliated deciduous tooth-mediated bone regeneration^12^. Furthermore, Yang *et al*. reported that Wnt3a, a representative canonical member of the Wnt family of ligands, upregulates the expression of β-catenin and enhances the osteogenic differentiation of dental follicle cells, while dickkopf1, a Wnt/β-catenin signalling inhibitor, decreases the expression of β-catenin and attenuates the osteogenic differentiation of dental follicle cells^13^. However, it remains unknown whether the Wnt/β-catenin pathway also participates in the osteogenic differentiation of EMSCs, which are the progenitor cells of dental mesenchymal stem cells.

In the present study and for the first time, we demonstrated that the Wnt/β-catenin pathway positively regulates EMSC osteogenic differentiation. Moreover, the Wnt/β-catenin pathway was targeted by low-affinity nerve growth factor receptor (LNGFR) during EMSC osteogenic differentiation. Additionally, LNGFR promoted EMSC osteogenic differentiation, which was decreased by the Wnt signalling inhibitor IWR-1-endo. Taken together, our data revealed that LNGFR targets the Wnt/β-catenin pathway and positively regulates EMSC osteogenic differentiation, suggesting that the Wnt/β-catenin pathway may be involved in tooth development and that targeting the Wnt/β-catenin pathway may have great potential for applications in dental tissue engineering regeneration.

## Results

### Isolation and identification of rat ectomesenchymal stem cells (EMSCs)

**Figure 1:**
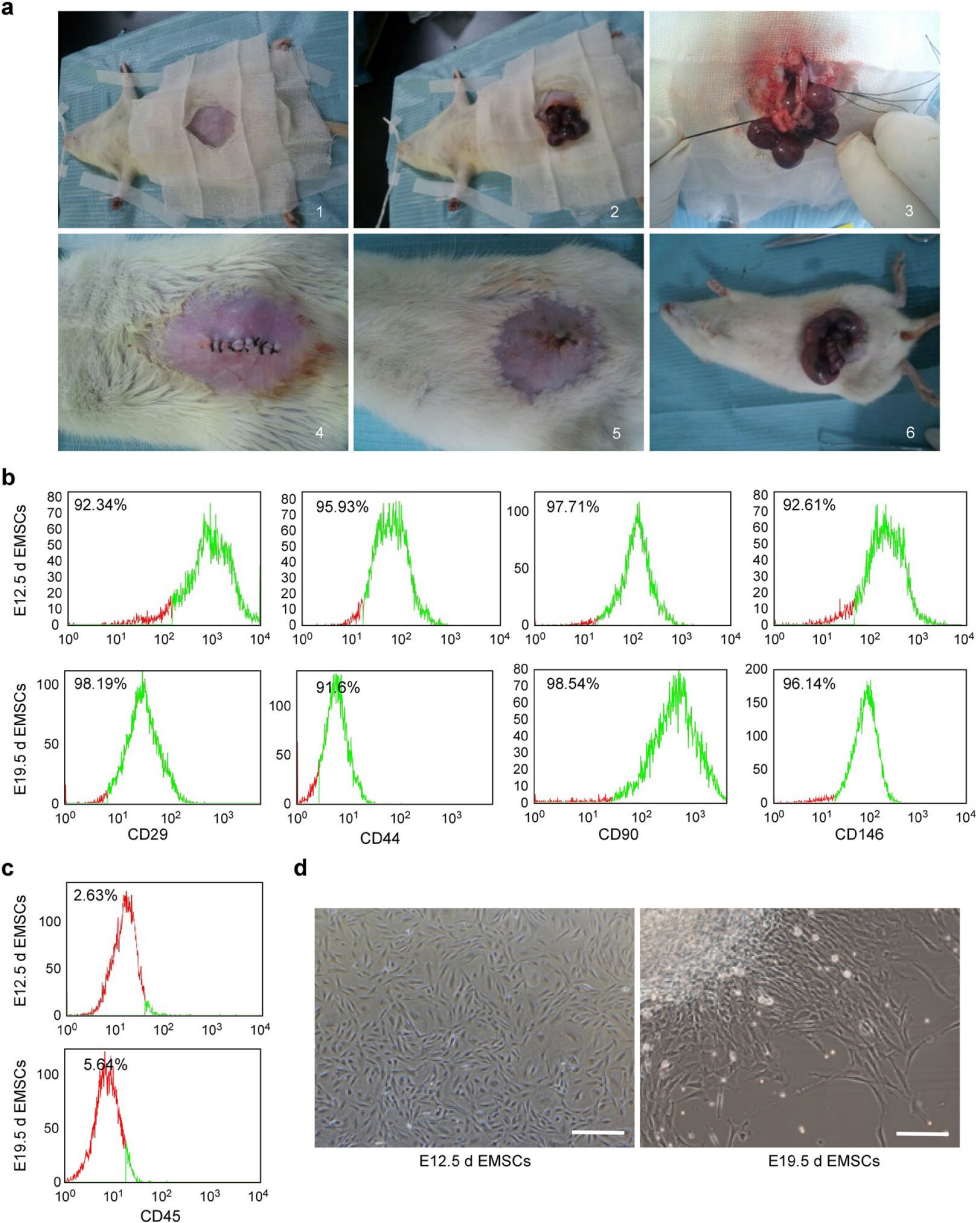
The isolation and identification of rat ectomesenchymal stem cells (EMSCs). (**a**–**d**) We isolated EMSCs from embryonic facial processes on day 12.5 and day 19.5 of the embryonic development process though an abdominal operation on the same pregnant SD rat. (**a**) The abdominal surgery steps are shown in pictures 1–6 The surgery steps in 1–4 were completed on embryonic day 12.5 (E12.5d), and those in 5–6 were completed on embryonic day 19.5 (E19.5d) on the same rat. (1) The SD pregnant rat was intraperitoneally injected with 2.5% phenobarbital sodium (1 ml/100 g) on day 12.5 of embryonic development and was then placed on a rat plate with towels for hair removal and disinfection. (2) Subsequently, the abdomen skin was cut along the abdominal midline, the abdominal muscles were separated, the abdominal cavity was exposed and the embryos were removed from the abdominal cavity. (3) The proximal, distal and mesenteric vessels of the embryos were ligated and the embryos were isolated. (4) Erythromycin (1 ml) and levofloxacin (1 ml) were dropped into the abdominal cavity and the muscle and skin were hierarchically sutured. (5) The same SD pregnant rat continued to grow to day 19.5 of embryonic development; by then, the incision had healed well. (6) The SD pregnant rat was then intraperitoneally injected with 2.5% phenobarbital sodium and was executed to obtain the remaining embryos. (**b**) The mesenchymal stem cell surface markers (CD29, CD44, CD90 and CD146) and (**c**) the haematopoietic marker CD45 were detected on EMSCs by the Flow cytometry. (**d**) The morphologies of the EMSCs separated on E12.5d and E19.5d were observed by optical microscopy. The scale bar represents 50 μm. E12.5d EMSCs, ectomesenchymal stem cells separated at E12.5d and E19.5d EMSCs, ectomesenchymal stem cells separated at E19.5d.

At 12.5 and 19.5 days of the embryonic development process, we isolated EMSCs though an abdominal operation on the same Sprague-Dawley (SD) pregnant rat (Fig. 1a). Our previous reports showed that EMSCs migrated to facial processes on day 11.5 of embryonic development and were in the bud stage on day 12.5 and in late bell stage on day 19.5^1^. The bud stage is the initiation stage of tooth germ development, at which tooth germ cells are not yet differentiated and the tooth germ is in morphogenesis; the late bell stage is the maturation stage of tooth germ development, at which the tooth germ was undergoing tissue differentiation and morphological differentiation. The bud and late bell stages represent the early and late stages of tooth germ development, respectively, which is why we chose these two time points to complete our experiments. Subsequently, we detected EMSC cell surface markers; the mesenchymal stem cell markers CD29, CD44, CD90 and CD146 were highly expressed in both EMSCs separated at embryonic day 12.5 (E12.5d) and embryonic day 19.5 (E19.5d) (Fig. 1b), while the haematopoietic marker CD45 was lowly expressed in both EMSCs separated at E12.5d and E19.5d (Fig. 1c). We also observed the morphology of the EMSCs by optical microscopy. The EMSCs showed a long spindle morphology, which is the morphological characteristic of mesenchymal stem cells (Fig. 1d). For the EMSCs separated at E19.5d, the highlighted area on the upper left corner was the tissue block, while for the EMSCs separated at E12.5d, there were no highlighted areas. Because EMSCs separated at E12.5d were obtained from the embryonic facial processes of E12.5d embryos, the tissues were soft and could be cut into very small organizations. Trypsin digestion and filtration were applied to remove the tissues and to obtain the EMSCs separated at E12.5d. EMSCs separated at E19.5d were obtained from the dental papilla of E19.5d embryos; the tooth germ tissues were relatively tough and were difficult to cut into small organizations. We cultured the tissue block to obtain the EMSCs separated at E19.5d rather than using a filtration method. During the culture of the tissue block, EMSCs separated at E19.5d radially climbed out from the tissue, leading to the uneven distribution. These results indicate that EMSCs isolated from the embryonic facial processes are indeed rat ectomesenchymal stem cells from the cranial nerve crest.

### Nuclear β-catenin is upregulated during EMSC osteogenic differentiation

**Figure 2:**
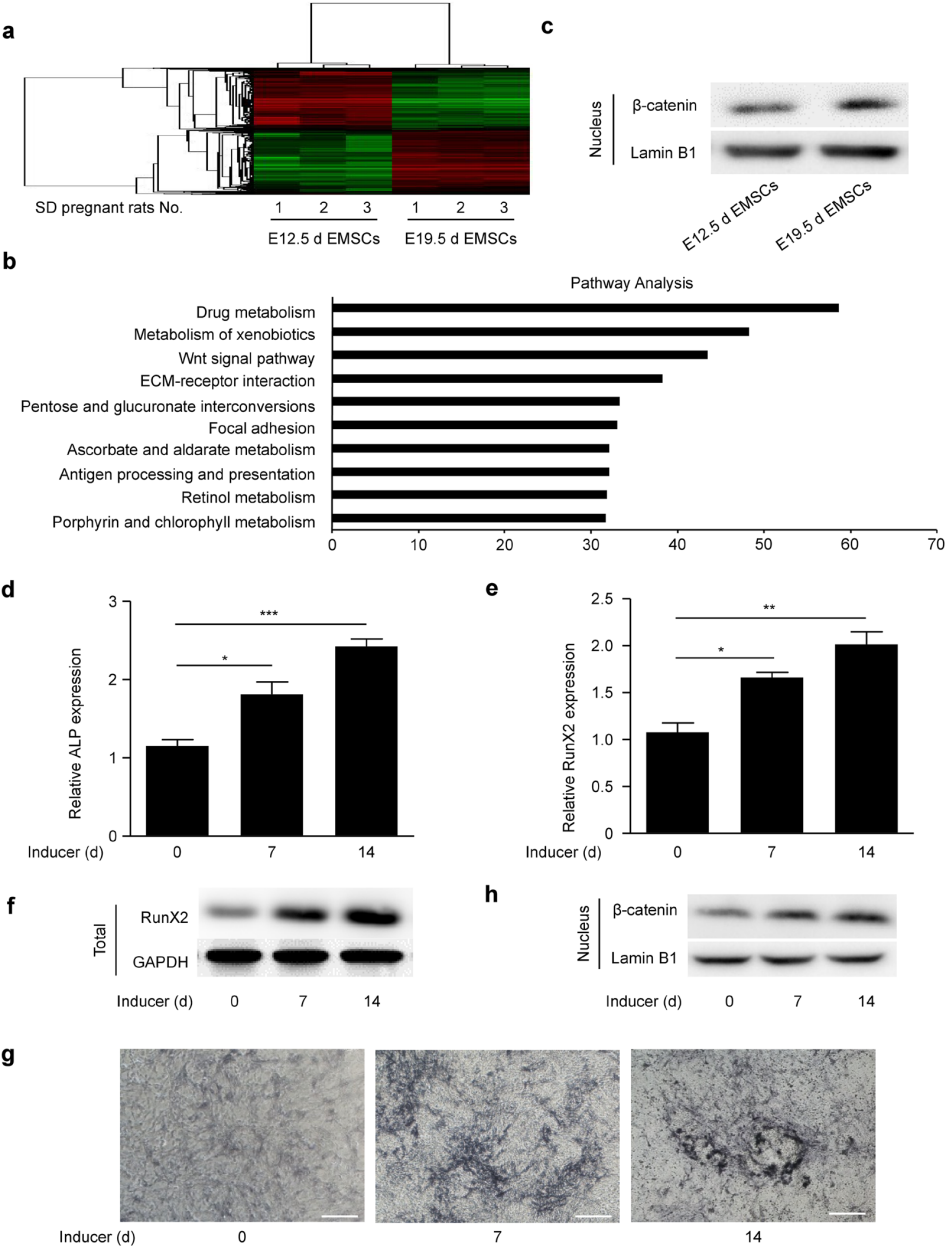
Nuclear β-catenin is upregulated during EMSC osteogenic differentiation. (**a**) Clustering analysis showed the differentially expressed genes between EMSCs separated at E12.5d and EMSCs separated at E19.5d. (**b**) Pathway analysis shows the pathways that the differentially expressed genes are involved in. (**c**) Nuclear β-catenin protein levels in EMSCs separated at E12.5d and E19.5d were detected by Western blot. Full-length blots are presented in Supplementary Figure S1a. (**d**–**h**) EMSCs separated at E19.5d were treated with osteogenic induction solution for 14 days. The osteogenic induction solution was changed every three days. The cells were harvested on days 0, 7 and 14 during osteogenic induction. Subsequently, (**d**) ALP and (**e**) RunX2 mRNA levels were detected by qPCR, (**f**) RunX2 protein levels were detected by Western blot, (**g**) the ALP staining depth was observed by optical microscopy, and (**h**) nuclear β-catenin protein levels in EMSCs were detected by Western blot. The full-length blots of (**f**) and (**h**) are presented in Supplementary Figure S1b and c, respectively. GAPDH was used as a total protein control and Lamin B1 was used as a nuclear protein control in Western blot. GAPDH was used as a control in qPCR. Scale bar represents 50 μm. E12.5d EMSCs, ectomesenchymal stem cells separated at E12.5d; E19.5d EMSCs, ectomesenchymal stem cells separated at E19.5d; ALP staining, alkaline phosphatase staining; and Inducer, the osteogenic induction solution. The results represent the mean ± SD from three independent experiments performed in triplicate. *P < 0.05, **P < 0.01, and ***P < 0.001.

To investigate the differentiation mechanism of EMSCs, we used an Affymetrix expression profiling microarray to detect the differences in gene expression in EMSCs separated at E12.5d and E19.5d; the results showed that 1105 genes were differentially expressed in the cluster analysis (Fig. 2a). Pathway analysis showed that the differentially expressed genes were highly involved in the Wnt signalling pathway (Fig. 2b). Therefore, we detected the nuclear levels of β-catenin in EMSCs separated at E12.5d and E19.5d. The results showed that nuclear β-catenin protein levels in EMSCs were higher at E19.5d compared to E12.5d (Fig. 2c). Subsequently, osteogenic induction solution was used to induce *in vitro* osteogenic differentiation of EMSCs separated at E19.5d, which were used in the following experiments. We found that the mRNA levels of the early osteogenic markers ALP and RunX2 were upregulated during osteogenic induction by qPCR assay (Fig. 2d and e). Similar results were observed for RunX2 protein levels by Western blot (Fig. 2f). Additionally, the ALP staining depth increased after osteogenic induction (Fig. 2g). These results showed that EMSCs have the potential to differentiate upon treatment with osteogenic induction solution. Thereafter, we detected the nuclear levels of β-catenin protein during EMSC osteogenic differentiation. The results showed that the nuclear levels of β-catenin protein are upregulated during osteogenic induction (Fig. 2h). Collectively, these results indicate that nuclear β-catenin levels are upregulated during EMSC osteogenic differentiation, suggesting that the Wnt/β-catenin pathway may be involved in regulating osteogenic differentiation in EMSCs.

### Wnt/β-catenin pathway positively regulates osteogenic differentiation in EMSCs

**Figure 3:**
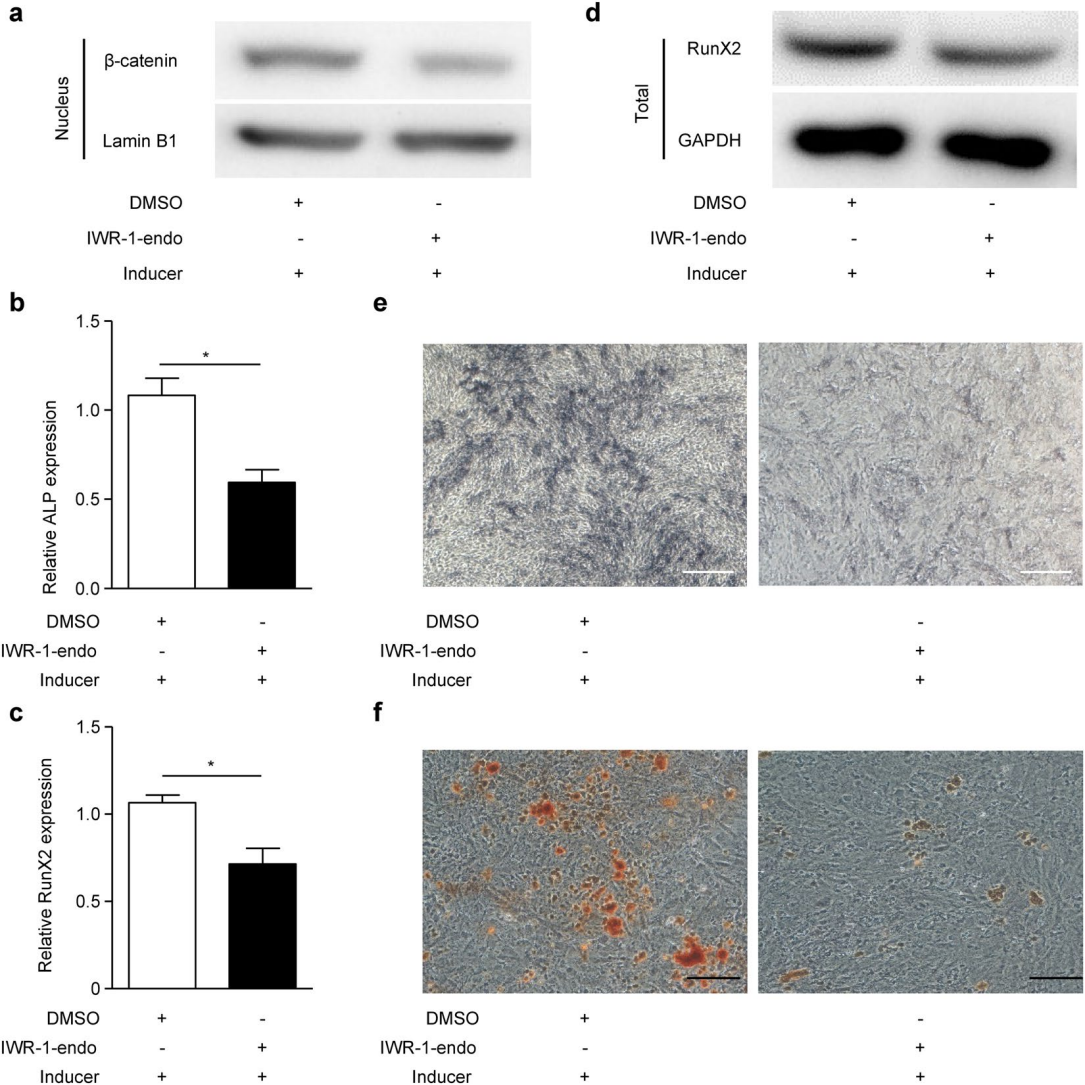
Wnt signalling inhibitor IWR-1-endo inhibits EMSC osteogenic differentiation. (**a**–**e**) EMSCs were pre-treated with the Wnt signalling inhibitor IWR-1-endo (10 µM) or DMSO for 24 h, and then treated with osteogenic induction solution for another 7 days. On day 3, the osteogenic induction solution was changed and IWR-1-endo (10 µM) was added. On day 7, the cells were harvested. Then, (**a**) nuclear β-catenin protein levels were detected by Western blot, (**b**) ALP and (**c**) RunX2 mRNA levels were detected by qPCR, (**d**) RunX2 total protein was detected by Western blot, and (**e**) ALP staining depth was observed by optical microscopy. Full-length blots of (**d**) are presented in Supplementary Figure S2a. (**f**) EMSCs were pre-treated with IWR-1-endo (10 µM) or DMSO for 24 h, and then treated with osteogenic induction solution for another 21 days. The osteogenic induction solution was changed every three days and IWR-1-endo (10 µM) was added each time. Then, the mineralized nodules were photographed after alizarin red staining. GAPDH was used as a total protein control and Lamin B1 was used as a nuclear protein control in Western blot. GAPDH was used as a control in qPCR. Scale bar represents 50 μm in ALP staining and 150 μm in alizarin red staining. ALP staining, alkaline phosphatase staining and Inducer, the osteogenic induction solution. The results represent the mean ± SD from three independent experiments performed in triplicate. *P < 0.05.

**Figure 4:**
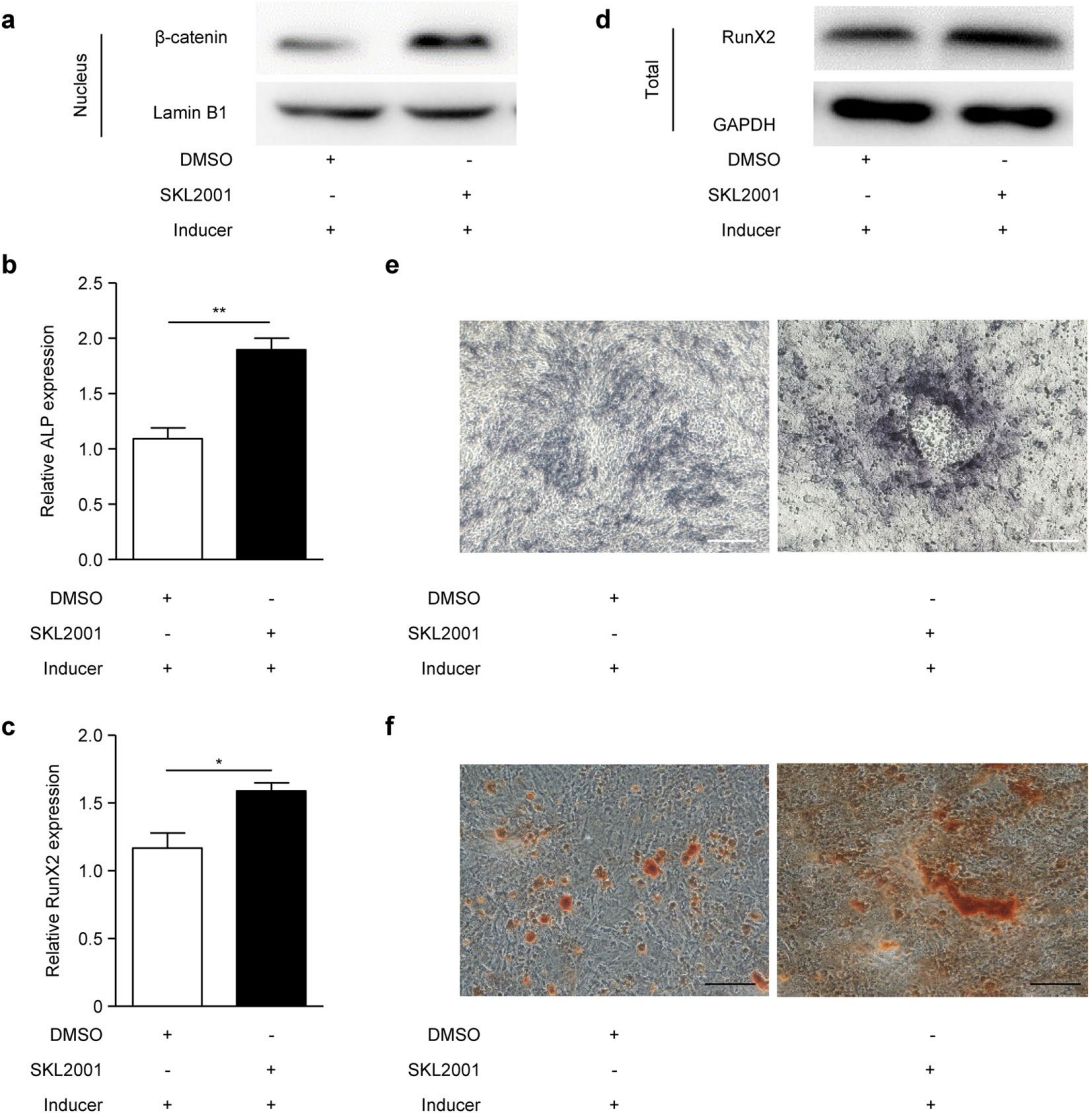
Wnt signalling agonist SKL2001 promotes EMSC osteogenic differentiation. (**a**–**e**) EMSCs were pre-treated with the Wnt signalling agonist SKL2001 (40 µM) or DMSO for 24 h, and then treated with osteogenic induction solution for another 7 days. On day 3, the osteogenic induction solution was changed and SKL2001 (40 µM) was added. On day 7, the cells were harvested. Then, nuclear (**a**) β-catenin protein levels were detected by Western blot, (**b**) ALP and (**c**) RunX2 mRNA levels were detected by qPCR, (**d**) RunX2 total protein was detected by Western blot, and (**e**) the ALP staining depth was observed by optical microscopy. Full-length blots of (**d**) are presented in Supplementary Figure S2b. (**f**) EMSCs were pre-treated with SKL2001 (40 µM) or DMSO for 24 h, and then treated with osteogenic induction solution for another 21 days. The osteogenic induction solution was changed every three days and SKL2001 (40 µM) was added each time. Then, the mineralized nodules were photographed after alizarin red staining. GAPDH was used as a total protein control and Lamin B1 was used as a nuclear protein control in Western blot. GAPDH was used as a control in qPCR. Scale bar represents 50 μm in ALP staining and 150 μm in alizarin red staining. ALP staining, alkaline phosphatase staining and Inducer, the osteogenic induction solution. The results represent the mean ± SD from three independent experiments performed in triplicate. *P < 0.05 and **P < 0.01.

To further study whether the Wnt/β-catenin pathway is involved in regulating EMSC osteogenic differentiation, we treated EMSCs with the Wnt signalling inhibitor IWR-1-endo and the Wnt signalling agonist SKL2001 before osteogenic induction. We found that IWR-1-endo markedly reduced the nuclear β-catenin protein levels during osteogenic differentiation in EMSCs (Fig. 3a). The results showed that IWR-1-endo decreased ALP and RunX2 mRNA levels during osteogenic differentiation in EMSCs (Fig. 3b and c). Similar results were observed for RunX2 protein levels by Western blot (Fig. 3d). Moreover, ALP staining depth decreased upon treatment with IWR-1-endo during osteogenic induction (Fig. 3e). Additionally, Alizarin red staining showed that the number of mineralized nodules decreased upon treatment with IWR-1-endo during osteogenic induction (Fig. 3f). Consistent with the above results, we found that SKL2001 increased the nuclear β-catenin protein levels (Fig. 4a), ALP and RunX2 mRNA levels (Fig. 4b and c), and RunX2 protein levels (Fig. 4d). Furthermore, ALP staining depth increased (Fig. 4e) and Alizarin red staining (Fig. 4f) showed more mineralized nodules upon treatment with SKL2001 during osteogenic induction. Taken together, these results suggest that the Wnt/β-catenin pathway positively regulates EMSC osteogenic differentiation.

### Overexpression of LNGFR upregulates nuclear β-catenin and enhances EMSC osteogenic differentiation

**Figure 5:**
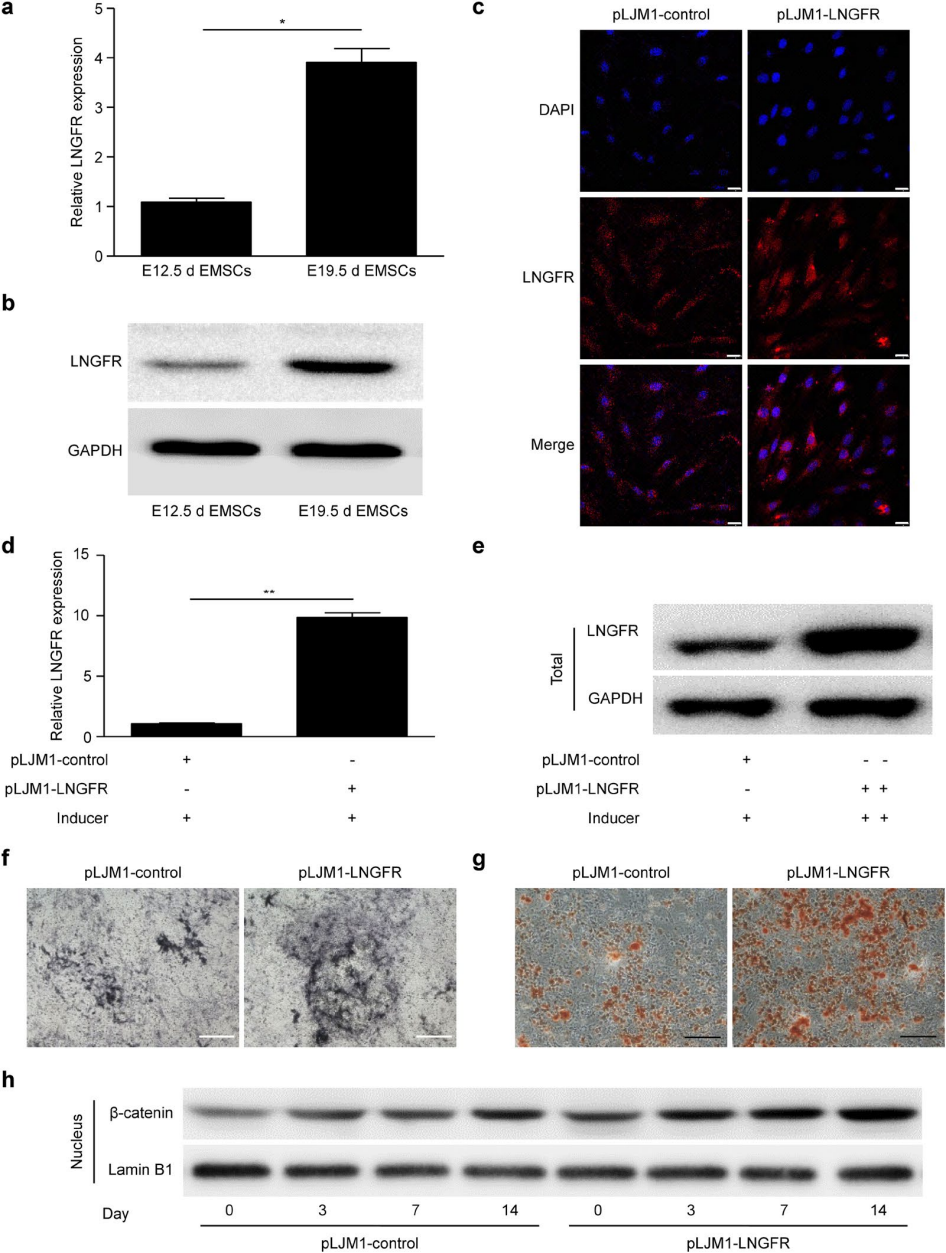
LNGFR overexpression increases nuclear β-catenin levels and promotes EMSC osteogenic differentiation. (**a**,**b**) The mRNA and protein levels of LNGFR in EMSCs separated at E12.5d and E19.5d were detected by (**a**) qPCR and (**b**) Western blot, respectively. Full-length blots of (**b**) are presented in Supplementary Figure S3a. (**c**) The levels of LNGFR in EMSCs stably overexpressing LNGFR and control EMSCs were detected by the immunofluorescence assays. Scale bar represents 50 μm. (**d**–**f** and **h**) EMSCs stably overexpressing LNGFR and control EMSCs were treated with osteogenic induction solution for 7 days. On day 3, the osteogenic induction solution was changed. On day 7, LNGFR mRNA and protein levels of LNGFR were detected by (**d**) qPCR and (**e**) Western blot, (**f**) the ALP staining depth was observed by optical microscopy (**f**), and (**h**) nuclear β-catenin protein levels were detected by Western blot. Full-length blots of (**e**) are presented in Supplementary Figure S3b. (**g**) EMSCs stably overexpressing LNGFR and control EMSCs were treated with osteogenic induction solution for 21 days. The osteogenic induction solution was changed every three days. Then, the mineralized nodules were photographed after alizarin red staining. GAPDH was used as a total protein control and Lamin B1 was used as a nuclear protein control in Western blot. GAPDH was used as a control in qPCR. Scale bar represents 50 μm in ALP staining and 150 μm in alizarin red staining. ALP staining, alkaline phosphatase staining; Inducer, the osteogenic induction solution; pLJM1-LNGFR, EMSCs stably overexpressing LNGFR; and pLJM1-control, control EMSCs. The results represent the mean ± SD from three independent experiments performed in triplicate. *P < 0.05 and **P < 0.01.

Low-affinity nerve growth factor receptor (LNGFR) is a receptor for neurotrophins and belongs to the tumour necrosis factor receptor (TNFR) superfamily^14^. Previous studies have showed that LNGFR is highly expressed in early developed nerve cells and participates in the process of embryogenesis including in proliferation, survival, apoptosis, differentiation, and migration^15–19^. We found that LNGFR expression is higher in EMSCs separated at E19.5d compared to EMSCs separated at E12.5d by qPCR assay (Fig. 5a) and Western blot (Fig. 5b). LNGFR expression was positively correlated with nuclear β-catenin levels, suggesting that LNGFR might be associated with β-catenin in promoting the osteogenic differentiation in EMSCs. Therefore, we tested the effects of LNGFR on nuclear β-catenin levels and EMSC osteogenic differentiation. Immunofluorescence assays showed that LNGFR expression was significantly upregulated in EMSCs stably overexpressing LNGFR compared to control EMSCs (Fig. 5c). qPCR assay (Fig. 5d) and Western blot (Fig. 5e) also revealed that LNGFR mRNA and protein levels were increased in EMSCs stably overexpressing LNGFR compared to control EMSCs during osteogenic induction. The ALP staining depth (Fig. 5f) increased and Alizarin red staining (Fig. 5g) showed more mineralized nodules in EMSCs stably overexpressing LNGFR compared to control EMSCs, suggesting that LNGFR promoted EMSC osteogenic differentiation. Moreover, we detected the β-catenin nuclear protein level of EMSCs stably overexpressing LNGFR and control EMSCs on days 0, 3, 7 and 14 during EMSC osteogenic differentiation. The results showed that β-catenin nuclear protein levels gradually increased in both EMSCs stably overexpressing LNGFR and control EMSCs during EMSC osteogenic differentiation, while the elevated tendency for elevated β-catenin was more obvious in EMSCs stably overexpressing LNGFR than in control EMSCs (Fig. 5h). These results indicate that LNGFR overexpression increases nuclear β-catenin levels and promotes EMSC osteogenic differentiation.

### LNGFR silencing downregulates nuclear β-catenin and weakens EMSC osteogenic differentiation

**Figure 6:**
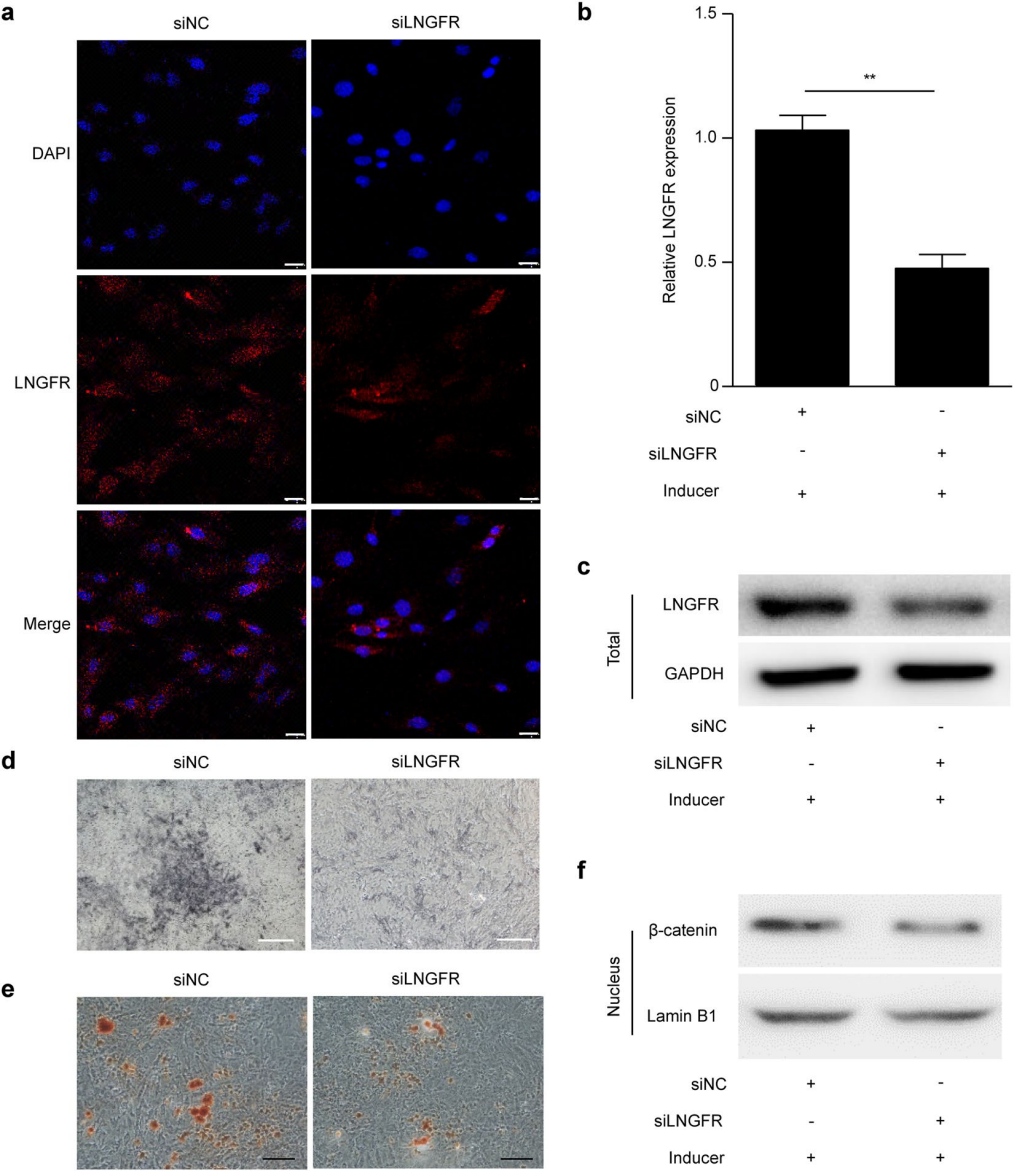
LNGFR silencing decreases nuclear β-catenin levels and decreased EMSC osteogenic differentiation. (**a**) EMSCs were transfected with siLNGFR or siNC for 24 h, and then LNGFR levels were detected in EMSCs by immunofluorescence assays. Scale bar represents 50 μm. (**b**–**d** and **f**) EMSCs, which had been transfected with siLNGFR or siNC for 24 h, were treated with osteogenic induction solution for another 7 days. On day 3, the osteogenic induction solution was changed. On day 7, the cells were harvested. Then, the mRNA and protein levels of LNGFR were detected by (**b**) qPCR and (**c**) Western blot, respectively, (**d**) the ALP staining depth was observed by optical microscopy, and (**f**) nuclear β-catenin protein levels were detected by Western blot. Full-length blots of (**c**) and (**f**) are presented in Supplementary Figure S4a and b, respectively. (**e**) EMSCs, which had been transfected with siLNGFR or siNC for 24 h, were treated with osteogenic induction solution for another 21 days. The osteogenic induction solution was changed every three days. Then, the mineralized nodules were photographed after alizarin red staining. GAPDH was used as a total protein control and Lamin B1 was used as a nuclear protein control in Western blot. GAPDH was used as a control in qPCR. Scale bar represents 50 μm in ALP staining and 150 μm in alizarin red staining. ALP staining, alkaline phosphatase staining; Inducer, the osteogenic induction solution; siLNGFR, siRNA for LNGFR; and siNC, negative control siRNA. The results represent the mean ± SD from three independent experiments performed in triplicate. *P < 0.05 and **P < 0.01.

We next silenced LNGFR during EMSC osteogenic differentiation. Immunofluorescence assays showed that LNGFR expression was significantly downregulated when LNGFR was silenced in EMSCs (Fig. 6a). qPCR assay (Fig. 6b) and Western blot (Fig. 6c) also revealed that LNGFR mRNA and protein levels decreased when LNGFR was silenced in EMSCs during osteogenic induction. The ALP staining depth (Fig. 6d) decreased and Alizarin red staining (Fig. 6e) showed fewer mineralized nodules when LNGFR was silenced in EMSCs, suggesting that LNGFR decreases EMSC osteogenic differentiation. Subsequently, we detected nuclear β-catenin levels and found that it decreased when LNGFR was silenced in EMSCs (Fig. 6f). These results indicate that silencing LNGFR decreases nuclear β-catenin levels and decreases EMSC osteogenic differentiation. Taken together, these results suggest that the Wnt/β-catenin pathway may be involved in LNGFR-enhanced EMSC osteogenic differentiation.

### LNGFR enhances EMSC osteogenic differentiation via targeting the Wnt/β-catenin pathway

**Figure 7:**
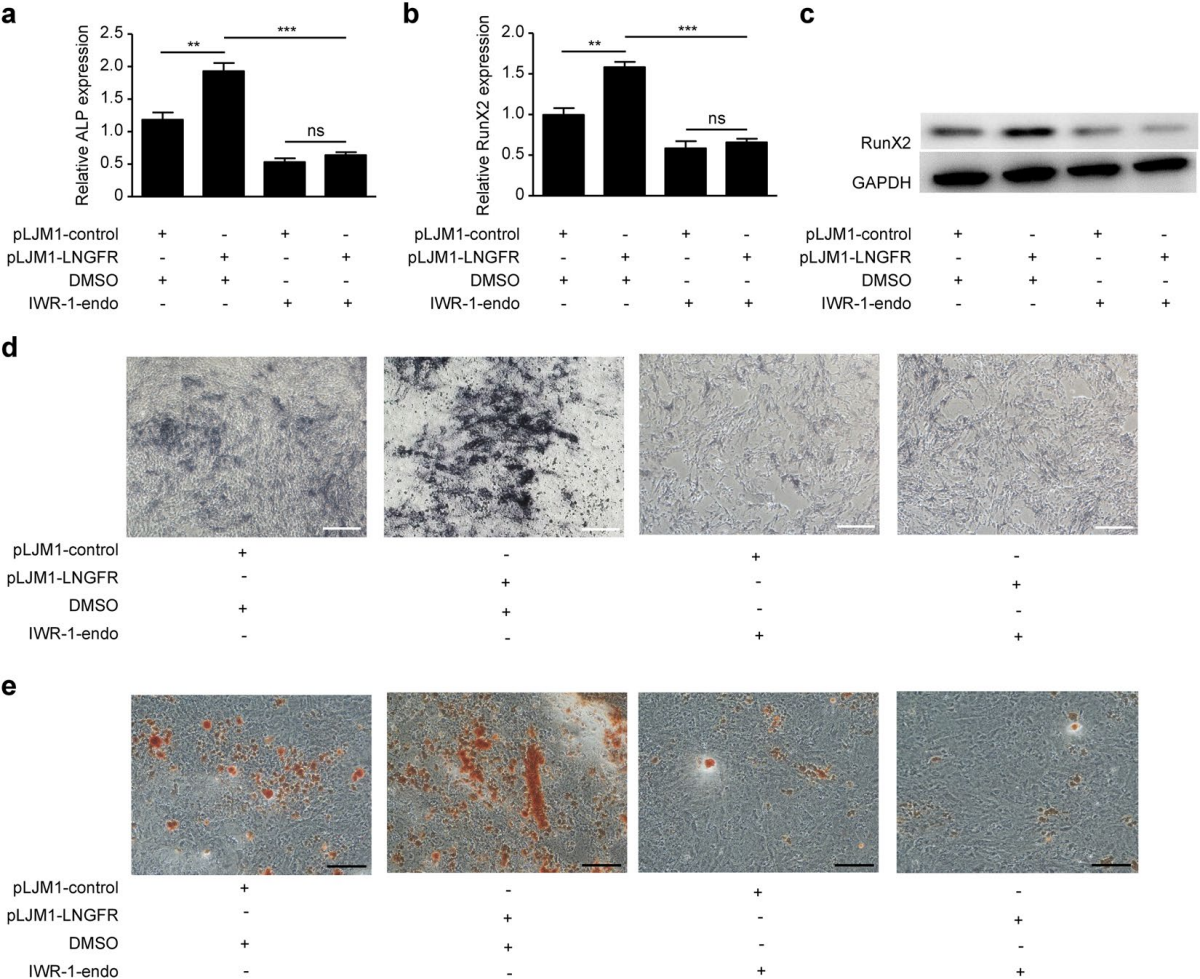
LNGFR enhances EMSC osteogenic differentiation by activating the Wnt/β-catenin pathway. (**a**–**d**) EMSCs stably overexpression LNGFR and control EMSCs were pre-treated with IWR-1-endo (10 µM) or DMSO for 24 h, and then treated with osteogenic induction solution for another 7 days. On day 3, the osteogenic induction solution was changed and IWR-1-endo (10 µM) was added. On day 7, the cells were harvested. Then, (**a**) ALP and (**b**) RunX2 mRNA levels were detected by qPCR, (**c**) RunX2 protein levels were detected by Western blot, and (**d**) the ALP staining depth was observed by optical microscopy. (**e**) EMSCs stably overexpression LNGFR and control EMSCs were pre-treated with IWR-1-endo (10 µM) or DMSO for 24 h, and then treated with osteogenic induction solution for another 21 days. The osteogenic induction solution was changed every three days and IWR-1-endo (10 µM) was added each time. The mineralized nodules were photographed after alizarin red staining. GAPDH was used as control both in qPCR and Western blot. Scale bar represents 50 µm in ALP staining and 150 μm in alizarin red staining. pLJM1-LNGFR, EMSCs stably overexpression LNGFR and pLJM1-control, control EMSCs. The results represent the mean ± SD from three independent experiments performed in triplicate. *P < 0.05, **P < 0.01, ***P < 0.001, and ns represents no significance.

To further study the role of the Wnt/β-catenin pathway in LNGFR-enhanced EMSC osteogenic differentiation, we inhibited the Wnt/β-catenin pathway using IWR-1-endo while overexpressing LNGFR during EMSC osteogenic differentiation. The results showed that without IWR-1-endo treatment LNGFR increased ALP and RunX2 mRNA level, while upon the treatment of IWR-1-endo, LNGFR no longer increased ALP and RunX2 mRNA levels (Fig. 7a and b). Similar results were observed for RunX2 protein levels by Western blot (Fig. 7c). Moreover, the ALP staining depth no longer increased upon IWR-1-endo treatment during osteogenic induction (Fig. 7d). Additionally, Alizarin red staining also showed that the number of mineralized nodules no longer increased upon IWR-1-endo treatment during osteogenic induction (Fig. 7e). Collectively, these results suggest that LNGFR enhances EMSC osteogenic differentiation at least in part by activating the Wnt/β-catenin pathway.

## Discussion

In this study, we demonstrate that nuclear β-catenin levels are higher in EMSCs separated at E19.5d compared to EMSCs separated at E12.5d and upregulated during EMSC osteogenic differentiation. Moreover, the Wnt signalling inhibitor IWR-1-endo inhibits, while the Wnt signalling agonist SKL2001 promotes EMSC osteogenic differentiation. Further experiments showed that nuclear β-catenin is further upregulated by LNGFR overexpression during EMSC osteogenic differentiation. Moreover, LNGFR promotes EMSC osteogenic differentiation, which is decreased by IWR-1-endo. Collectively, our data show that the LNGFR targets the Wnt/β-catenin pathway targeted and positively regulates osteogenic differentiation in EMSCs.

Low-affinity nerve growth factor receptor (LNGFR) is the receptor of neurotrophins, a family of protein growth factors that stimulate survival and differentiation in neuronal cells^20, 21^. In this study, we found that LNGFR expression is higher in EMSCs separated at E19.5d compared to EMSCs separated at E12.5d. Based on this result, we speculated that LNFGR might be involved in the regulation of osteogenic differentiation in EMSCs. We then constructed EMSCs stably overexpressing LNGFR and found that their potential for osteogenic differentiation was higher than control EMSCs. Prior to our study, emerging studies had reported that LNGFR is positively associated with osteogenic differentiation in other cell types. Alexander *et al*. reported that LNGFR is expressed at higher levels in mineralizing isolated jaw periosteum-derived cells (JPCs) compared with non-mineralizing JPCs^22^. Moreover, Akiyama *et al*. reported that LNGFR overexpression induced alkaline phosphatase activity and the mRNA expression of osteoblast-related genes such as osterix and bone sialoprotein, thus promoting osteoblast differentiation in the human MG63 osteoblast cell line^23^. Furthermore, Mikami *et al*. reported that LNGFR overexpression enhanced proliferation and osteogenic differentiation in the MC3T3-E1 pre-osteoblast cell line^18^. Regarding tooth development, current studies mainly focus on the role of LNGFR as a neural stem cell marker^24–26^. It remains to be determined whether LNGFR can regulate osteogenic differentiation in other dental mesenchymal stem cells.

The Wnt/β-catenin pathway can be regulated at several points in its signalling pathways^27^. These sites include the binding of extracellular ligands and cell membrane receptors, the stability of the destruction complex, and the co-transcriptional activation of β-catenin^28–33^. In this study, we used two small molecules (IWR-1-endo and SKL2001) to regulate the Wnt/β-catenin pathway. IWR-1-endo and SKL2001 both affect the function of the destruction complex, which contains Axin, protein phosphatase 2A (PP2A), adenomatosis polyposis coli (APC), glycogen synthase kinase 3 (GSK3) and casein kinase 1α (CK1α). We used IWR-1-endo to inhibit the Wnt/β-catenin pathway and found that IWR-1-endo decreased nuclear β-catenin levels and subsequently inhibited EMSC osteogenic differentiation. IWR-1-endo was first identified as an inhibitor of Wnt/β-catenin in 2009^34^ and reported to abrogate the destruction of Axin proteins, thus suppressing Wnt/β-catenin pathway activity^31^. Recent studies have used IWR-1-endo to inhibit the activity of Wnt/β-catenin^35–37^. Consistent with our research on cell differentiation, Frith *et al*. have reported that IWR-1-endo decreases the expression levels of key osteogenic marker genes and inhibits osteogenesis in mesenchymal precursor cells^38^. Moreover, Gao *et al*. have reported that IWR-1-endo indeed reduced the amounts of β-catenin in the nucleus, as well as TCF1, a β-catenin target gene, thus activating Wnt signalling, which is required for hepatocyte differentiation^39^. In this study, we also used a novel agonist of the Wnt/β-catenin pathway, SKL2001, during EMSC osteogenic differentiation. We found that SKL2001 increased nuclear β-catenin levels and subsequently enhanced EMSC osteogenic differentiation. SKL2001 is reported to increase the intracellular β-catenin protein levels by downregulating the interactions between Axin and β-catenin^28, 40^. Consistent with our study, Gwak *et al*. reported that the treatment of mesenchymal stem cells with SKL2001 promoted osteoblastogenesis by activating the Wnt/β-catenin pathway^28^. However, the roles of these two small molecules in the development of teeth require further study.

β-catenin, a protein that is encoded by the *CTNNB1* gene, is a dual function protein^32, 41^. On one hand, β-catenin is a subunit in the cadherin protein complex and is involved in regulating cell–cell adhesion. On the other hand, β-catenin is a downstream component of the Wnt signalling pathway and serves as a transcriptional co-activator^42–44^. The activation of Wnt signalling leads to a decrease in β-catenin degradation and a subsequent accumulation of β-catenin^32, 45^. In our experiments, nuclear β-catenin levels were further upregulated by LNGFR overexpression during EMSC osteogenic differentiation, suggesting that the Wnt/β-catenin pathway was targeted by LNGFR. However, targeting LNGFR in the Wnt/β-catenin pathway during EMSC osteogenic differentiation is not fully understood. A previous study showed that LNGFR bound to nerve growth factor (NGF) activates casein kinase II (CK2), which subsequently phosphorylates the phosphatase and tensin homologue deleted on chromosome 10 (PTEN)^46^. Phosphorylated PTEN becomes inactive and allows phosphatydilinositol 3-phosphate [PI(3)P] levels to increase, thus favouring GSK-3β inactivation and axon outgrowth in hippocampal neurons^46^. Another study showed that NGF fails to activate GSK3b phosphorylation of MAP1B in PC12 cells and Chick ciliary ganglion neurons that lack TrkA receptors but express LNGFR^47^. These two studies reveal that LNGFR is capable of promoting GSK-3β inactivation, which subsequently leads to the accumulation of β-catenin. However, in our stem cell model, LNGFR is unlikely to target the Wnt/β-catenin pathway and subsequently upregulate nuclear β-catenin by inactivating GSK-3β. We found that LNGFR-enhanced EMSC osteogenic differentiation was attenuated by IWR-1-endo, which promotes β-catenin degradation by abrogating Axin protein turnover and thereby enhances the function of the β-catenin destruction complex^36^. Considering the mechanism by which IWR-1-endo inhibits the Wnt/β-catenin pathway, we speculate that the points in the Wnt/β-catenin pathway targeted by LNGFR are located upstream of the β-catenin destruction complex. More studies are needed to understand a role of LNGFR in targeting the Wnt/β-catenin pathway during EMSC osteogenic differentiation.

EMSCs are the progenitor cells of dental mesenchymal stem cells. Therefore, it is important to investigate the mechanism of EMSC differentiation, which is helpful to improve our understanding of tooth development and provide a potential strategy for dental tissue engineering. In this paper, we found that the Wnt/β-catenin pathway positively regulates osteogenic differentiation in EMSCs, suggesting that the Wnt/β-catenin pathway is very important in early tooth development and that targeting the Wnt/β-catenin pathway may have great potential in tooth repair and dental tissue engineering regeneration.

## Materials and Methods

### Isolation of EMSCs and cell culture

E19.5d EMSCs and E12.5d EMSCs were separated during an abdominal operation on the same pregnant SD rat (provided by the Experimental Animal Centre of the Third Military Medical University). This study was approved by the Ethics Committee of the Third Military Medical University, and all experiments were performed in accordance with relevant guidelines and regulations. The operation was strictly performed under sterile operating conditions. E12.5d EMSCs were separated from the embryonic facial processes using 1% trypsin/1 mM EDTA (Sigma, St Louis, MO, USA) at 37 °C for 10 min. E19.5d EMSCs were separated from the dental papilla using 1% collagenase I for digestion at 37 °C for 30 min. The digestion was terminated using DMEM/F12 (Gibco, Waltham, MA, USA) containing 10% FBS (Gibco, Waltham, MA). E12.5d EMSCs were filtered to remove the tissue blocks using a 75 µm screen mesh (BD Biosciences, Franklin Lakes, NJ, USA). After centrifugation at 800 rpm for 5 min, E12.5d EMSCs were cultured in DMEM/F12 containing 10% FBS and antibiotics (100 µg/ml penicillin and 100 µg/ml streptomycin) in a 37 °C and 5% CO_2_ incubator. E19.5d EMSCs were obtained by directly culturing the tissue blocks in DMEM/F12 containing 10% FBS and antibiotics.

### Flow cytometry

The properties of the E19.5d EMSCs and E12.5d EMSCs were detected using flow cytometry as previously described^48^. Cell surface molecular markers, including CD29, CD44, CD45, CD90, and CD146, were detected in 3rd generation cells. A total of 5 × 10^5^ cells were fixed in 4% paraformaldehyde for 30 min and incubated in primary antibodies, including mouse anti-rat CD29, CD44, CD45, CD90 and CD146 (1:100; Santa Cruz Biotechnology, Santa Cruz, CA, USA), at 4 °C overnight. The secondary antibody anti-mouse IgG-FITC (1:1000) was then added, and the results were detected using a FACS Calibur flow cytometer (BD Biosciences).

### qRT-PCR detection

The expression of LNGFR, ALP, and RunX2 in E19.5d EMSCs and E12.5d EMSCs was detected using qRT-PCR as previously described^49^. The primer sequences are listed in Supplementary Table S1.

### Western blot assays

Cells were collected and the total protein and nuclear protein were extracted according to the instructions (Beyotime, P0013B and P0027, respectively). The protein concentration was measured using a BCA protein determination reagent kit (Beyotime, P0011). Western blotting was performed as previously described^50^. The primary antibodies included anti-GAPDH (YM1038) purchased from Immunoway; anti-LNGFR (ab8874) and anti-RunX2 (ab23981) purchased from Abcam; and anti-β-catenin (8480) and anti-Lamin B1 (13435) purchased from CST.

### Osteogenic induction in EMSCs

EMSCs were cultured in DMEM/F12 containing 10% foetal bovine serum (FBS). Third generation cells were used in the experiment. The osteogenic induction solution contained 10% FBS α-MEM (containing 50 mg/mL ascorbic acid, 10 mmol/L b-glycerol phosphate, and 100 nM dexamethasone). The cell culture medium was changed every 3 days.

### Alkaline phosphatase staining

On day 7 of osteogenic induction, the cells in each well were washed twice with PBS and fixed in 4% paraformaldehyde for 30 min. Staining was performed using an ALP staining kit (Beyotime, Shanghai, China) according to the manufacturer’s instructions.

### Alizarin red staining

On day 21 of osteogenic induction, the cells in each well were washed twice with PBS and fixed in 4% paraformaldehyde for 30 min. Alizarin red staining was performed as previously described^51^. After staining, the cells were washed 3 times with double-distilled water and the staining levels were observed under a phase-contrast microscope.

### LNGFR silencing using siRNA

The 3rd generation E19.5d EMSCs were plated onto 6-well plates at 70–90% confluence and were cultured for 24 h. Two hours before transfection, the culture medium was changed to DMEM/F12 (without penicillin, streptomycin, and FBS). siRNA-LNGFR and siRNA-NC (GenePharma, Shanghai, China) were transfected into E19.5d EMSCs using Lipofectamine 2000 in OPI-MEM (Invitrogen, Carlsbad, CA, USA). The siRNA sequences are listed in Supplementary Table S2.

### The construction of the EMSCs stably overexpressing LNGFR

The pLJM1-LNGFR plasmid and psPAX2 and CMV VSV-G packaging plasmids^52^ were co-transfected into HEK-293 cells using Lipofectamine 2000 (Invitrogen) as previously described^53^. Twenty-four hours after transfection, the supernatant containing packaged viruses was collected and filtered before use. The 3rd generation EMSCs were infected using 8 μg/mL polybrene. After 24 h, the cells were cultured in DMEM/F12 containing 2 μg/mL puromycin for one week.

### Immunofluorescence assays

The EMSCs stably overexpressing LNGFR, control EMSCs, EMSCs transfected with siRNA-LNGFR and EMSCs transfected with siRNA-NC were inoculated onto cover slips overnight. The cells were fixed in 4% paraformaldehyde and incubated with rabbit anti-rat LNGFR (1:100) and goat anti-mouse or anti-rabbit IgG-TRITC (red fluorescence) secondary antibodies. The cells were then stained with DAPI (Sigma) and observed under a confocal laser scanning microscope (TCS SP2; Leica Microsystems, Heidelberg, Germany).

### Microarray detection and bioinformatics analysis

Total RNA was extracted from the cells using the Trizol reagent (Invitrogen) according to the manufacturer’s instructions. Any contaminating deoxyribonucleic acids were removed via DNase I digestion. The Affymetrix GeneChip Rat Genome 230 2.0 Array was used for the microarray analysis. Three independent cell samples were used in the microarray analysis (n = 3). Hybridization, data acquisition, and analyses were performed by the CapitalBio Corporation (Beijing, China). The microarray results were analysed using the robust multiarray average (RMA) method. Significance analysis of microarrays (SAM) was used to identify differentially expressed genes. SAM-identified genes with statistically significant expression changes using a set of gene-specific t-tests and a permutation process provided an estimated false discovery rate (FDR) from the generated data. Genes with scores higher than the threshold value or genes with FDR values lower than the threshold value were considered significant. Additionally, fold-change analysis was performed to calculate the ratios of the geometric means of expression intensities. To select differentially expressed genes, we used ≥1.5**-** and ≤1.5-fold changes as threshold values and a significance level of <5% FDR. Signalling pathway analyses were performed for the genes with significant differences using the DAVID online analysis tool.

### Statistical analysis

All the data are expressed as the means ± standard deviations. Statistical significance was evaluated using the Prism 5 software. Comparisons were performed using t**-**tests and one**-**way analysis of variance (ANOVA). All the experiments were repeated in triplicate to ensure that the results were real and reliable. *P* < 0.05 indicated statistical significance.

## Electronic supplementary material

Supplementary Information
