# Supplementary material for: LNGFR targets the Wnt/β-catenin pathway and promotes the osteogenic differentiation in rat ectomesenchymal stem cells
Source: Sci Rep. 2017 Sep 8;7:11021. doi: 10.1038/s41598-017-11555-9 (PMC5591262; doi:10.1038/s41598-017-11555-9)
Supplement: Supplementary file 1 — Supplementary Information [file 41598_2017_11555_MOESM1_ESM.doc]

**LNGFR targets the Wnt/β-catenin pathway and promotes the osteogenic differentiation in rat ectomesenchymal stem cells**

Gang Li,^1^ Junyu Liu,^1^ Yingying Wang,^1^ Kun Yang,^2^ Manzhu Zhao,^1^Yong Xiao,^1^Xiujie Wen,^1^ Luchuan Liu^1^

**Supplementary Table S1. Primers for quantitative real-time PCR**

| GENE | Forward 5′—3′ | Reverse 5′—3′ |
| --- | --- | --- |
| LNGFR | CAACTTGGGCGAAGGCGT | ACACAGGGAGCGGACATGC |
| RunX2 | AGCGGACGAGGCAAGAGTTT | CTGTCTGTGCCTTCTTGGTTCC |
| ALP | GGCTCTGCCGTTGTTTCTCT | AAGGTGCTTTGGGAATCTGC |
| β-catenin  RatGAPDH | TGATAAAGGCAACTGTTGGATTGA  AAGTTCAACGGCACAGTCAAGG | CCGCTGGGTGTCCTGATGT  ACGCCAGTAGACTCCACGACAT |

**Supplementary Table S2. Sequences of the siRNA**

| product | Forward 5′—3′ | Reverse 5′—3′ |
| --- | --- | --- |
| siLNGFR (1145) | GCGUGAGGAGGUAGAGAAATT | UUUCUCUACCUCCUCACGCTT |
| siNC | UUCUCCGAACGUGUCACGUTT | ACGUGACACGUUCGGAGAATT |

**Supplementary Figure 1**


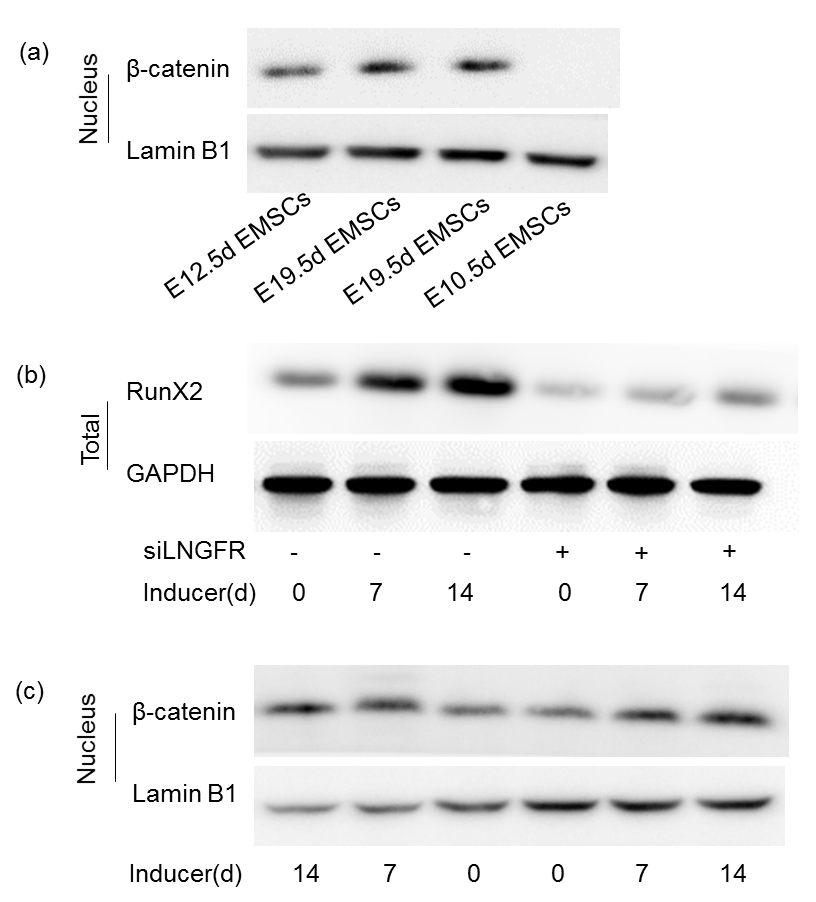


**Figure S1. The nuclear β-catenin is upregulated during the osteogenic differentiation of EMSCs. (a)** The nuclear protein levels of β-catenin in EMSCs separated at 10.5th day, 12.5th day and 19.5th day were detected by Western blot. **(b)** EMSCs separated at 19.5th day were transfected with siLNGFR/siNC for 24 h, and then treated with the osteogenic induction solution for another 14 days. The osteogenic induction solution was changed every three days. On day 0, day 7 and day 14 during the osteogenic differentiation, the cells were harvested. Subsequently, the protein level of RunX2 was detected by Western blot. **(c)** EMSCs separated at 19.5th day were treated with the osteogenic induction solution for 14 days. The osteogenic induction solution was changed every three days. On day 0, day 7 and day 14 during the osteogenic differentiation, the cells were harvested. Subsequently, the nuclear protein level of β-catenin were detected by Western blot. E10.5d EMSCs: ectomesenchymal stem cells separated at 10.5th day; E12.5d EMSCs: ectomesenchymal stem cells separated at 12.5th day; E19.5d EMSCs: ectomesenchymal stem cells separated at 19.5th day; siLNGFR: siRNA for LNGFR; siLNGFR/siNC: siLNGFR or siNC; Inducer: the osteogenic induction solution.

**Supplementary Figure 2**


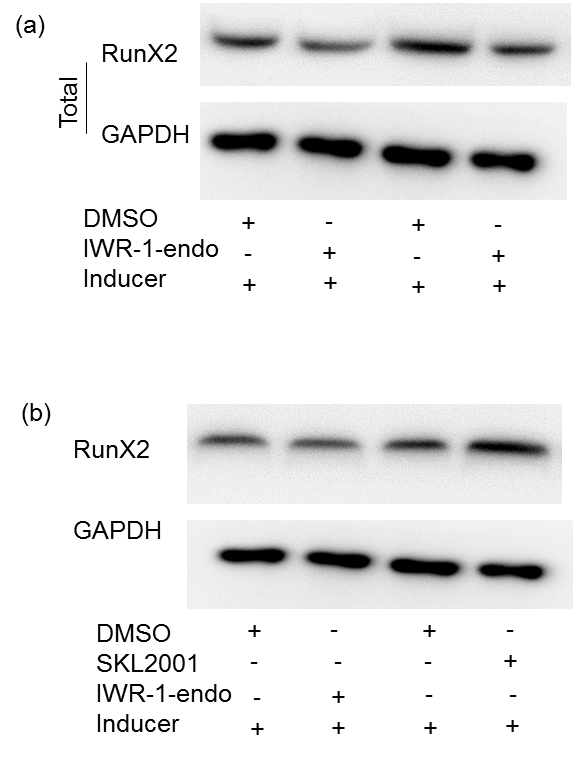


**Figure S2. Wnt signaling pathway positively regulates the osteogenic differentiation of EMSCs. (a)** EMSCs were pre-treated with the Wnt signaling inhibitor IWR-1-endo (10µM)/DMSO for 24 h, then treated with the osteogenic induction solution for another 7 days. On day 3, the osteogenic induction solution was changed and IWR-1-endo (10µM) was added. On day 7, the cells were harvested. Then the total protein of RunX2 was detected by Western blot. **(b)** EMSCs were pre-treated with the Wnt signaling inhibitor IWR-1-endo (10µM)/DMSO and Wnt signaling activator SKL2001 (40µM)/DMSO for 24 h, then treated with the osteogenic induction solution for another 7 days. On day 3, the osteogenic induction solution was changed, and IWR-1-endo (10µM)/SKL2001 (40µM) was added. On day 7, the cells were harvested. Then the total protein of RunX2 was detected by Western blot. IWR-1-endo (10µM)/DMSO: IWR-1-endo (10µM) or DMSO; SKL2001 (40µM)/DMSO: SKL2001 (40µM) or DMSO; IWR-1-endo (10µM)/SKL2001 (40µM): IWR-1-endo (10µM) or SKL2001 (40µM); Inducer: the osteogenic induction solution.

**Supplementary Figure 3**


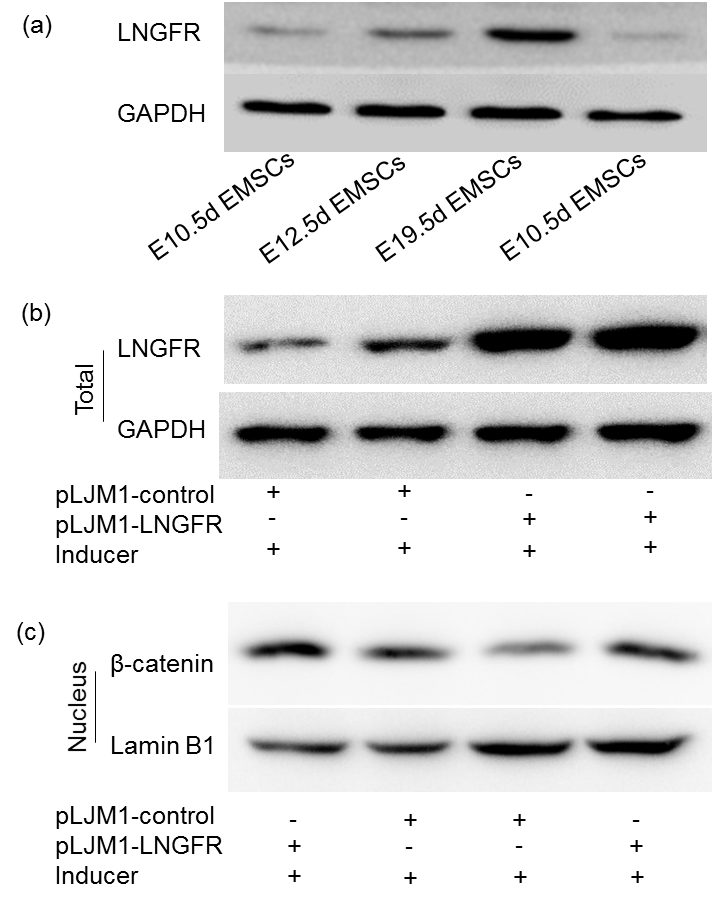


**Figure S3. The protein level of LNGFR is upregulated during the embryonic development and after overexpression of LNGFR. (a)** The protein level of LNGFR in EMSCs separated at 10.5th day, 12.5th day and 19.5th day were detected by Western blot. **(b)** LNGFR-stably overexpressed EMSCs and control EMSCs were treated with the osteogenic induction solution for 7 days. On day 3, the osteogenic induction solution was changed. On day 7, the cells were harvested. Then the protein level of LNGFR was detected by Western blot. E10.5d EMSCs: ectomesenchymal stem cells separated at 10.5th day; E12.5d EMSCs: ectomesenchymal stem cells separated at 12.5th day; E19.5d EMSCs: ectomesenchymal stem cells separated at 19.5th day; pLJM1-LNGFR: LNGFR-stably overexpressed EMSCs; pLJM1-control: control EMSCs; Inducer: the osteogenic induction solution.

**Supplementary Figure 4**


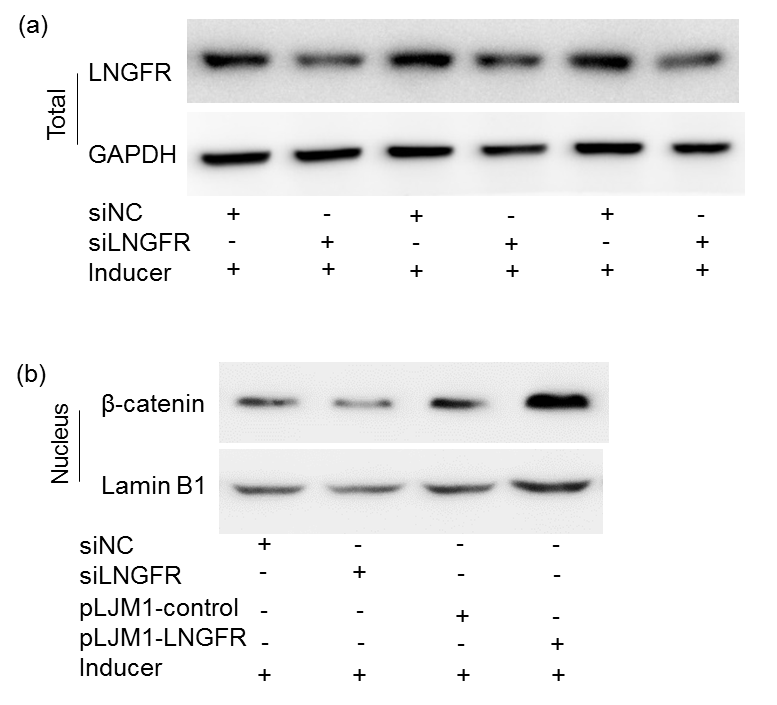


**Figure S4. LNGFR positively regulates the nuclear level of β-catenin. (a)** EMSCs were transfected with siLNGFR/siNC for 24 h, then treated with the osteogenic induction solution for another 7 days. On day 3, the osteogenic induction solution was changed. On day 7, the cells were harvested. Then protein level of LNGFR was detected by Western blot. **(b)** EMSCs were transfected with siLNGFR/siNC or pLJM1-LNGFR/pLJM1-control for 24 h, then treated with the osteogenic induction solution for another 7 days. On day 3, the osteogenic induction solution was changed. On day 7, the cells were harvested. Then the nuclear protein level of β-catenin was detected by Western blot. siLNGFR: siRNA for LNGFR; siNC, negative control siRNA; pLJM1-LNGFR: LNGFR-stably overexpressed EMSCs; pLJM1-control: control EMSCs; siLNGFR/siNC: siLNGFR or siNC; pLJM1-LNGFR/pLJM1-control: pLJM1-LNGFR or pLJM1-control; Inducer: the osteogenic induction solution.
